# Islands of Conformational Stability for Filopodia

**DOI:** 10.1371/journal.pone.0059010

**Published:** 2013-03-21

**Authors:** D. Robert Daniels, Matthew S. Turner

**Affiliations:** 1 Multidisciplinary Nanotechnology Centre, College of Engineering, Swansea University, Swansea, United Kingdom; 2 Department of Physics and Centre for Complexity Science, University of Warwick, Coventry, United Kingdom; University of Cambridge, United Kingdom

## Abstract

Filopodia are long, thin protrusions formed when bundles of fibers grow outwardly from a cell surface while remaining closed in a membrane tube. We study the subtle issue of the mechanical stability of such filopodia and how this depends on the deformation of the membrane that arises when the fiber bundle adopts a helical configuration. We calculate the ground state conformation of such filopodia, taking into account the steric interaction between the membrane and the enclosed semiflexible fiber bundle. For typical filopodia we find that a minimum number of fibers is required for filopodium stability. Our calculation elucidates how experimentally observed filopodia can obviate the classical Euler buckling condition and remain stable up to several tens of 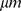. We briefly discuss how experimental observation of the results obtained in this work for the helical-like deformations of enclosing membrane tubes in filopodia could possibly be observed in the acrosomal reactions of the sea cucumber *Thyone*, and the horseshoe crab *Limulus*. Any realistic future theories for filopodium stability are likely to rely on an accurate treatment of such steric effects, as analysed in this work.

## Introduction

Filopodia are formed by the growth of bundles of biological fibers outwards from a biological cell surface that remain enclosed in a membrane tube. They are implicated in many processes vital to life, including sensing and motility [1] [2] [3]. There has therefore been much interest recently in the formation and growth of long, thin cellular protrusions due to the polymerization of bundles of fibers, including actin [4]. Such structures appear on cell membranes as familiar filopodia [1] [4], but can also appear on neural growth cones [5], sickled red blood cells [6] [7], the acrosomal reaction of the sea cucumber *Thyone* [8] [9], as well as on vesicles observed in vitro [10].

In this work, we investigate the stability of filopodia, which involves the subtle interplay between a fluid membrane tube, and an enclosed semiflexible fiber bundle. The simplest physical picture of filopodia is one in which the membrane tube produces a longitudinal force and a transverse force on the enclosed fiber bundle. The longitudinal membrane force acts to try and shorten the end-to-end distance of the fiber bundle, while the transverse force is required to maintain fiber bundle enclosure. The energetics required to investigate the stability of filopodia thus necessitates us to consider the elasticity of both the membrane tube as well as the fiber bundle, subject to the constraint that the polymer bundle must remain enclosed by the membrane tube. The energetic ground state conformations of filopodia thus necessitate a careful theoretical treatment of both elastic and steric considerations. For example, one might ask if a filopodium ever buckles, or perhaps more intriguingly does the region of filopodium buckling exist in some small corner of a complicated energetic phase diagram, well outside the range of physiologically relevant parameters?

A naive Euler buckling type estimate for the stability of filopodia [11] [12] suggests a limiting length of 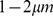. Additionally, the presence of cross-linking in the fiber bundle, and hence increased stiffness, further suggest a limiting length of 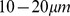 for stable filopodia [11] [12]. However, filopodia many tens of 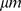 have been observed experimentally [8] [13].

**Figure 1:**
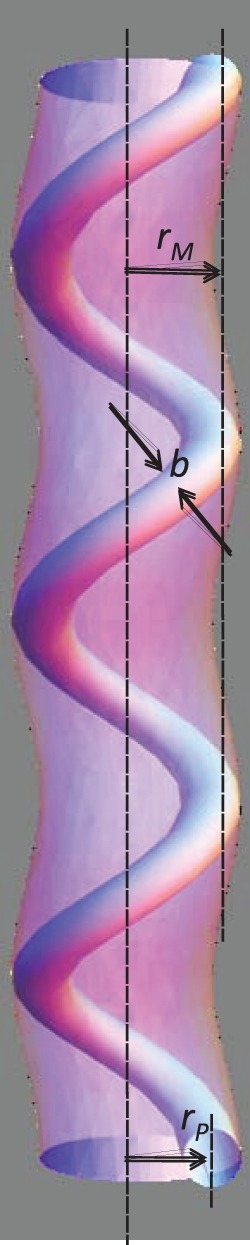
Sketch of a helically deformed membrane enclosing a helical fiber bundle. The membrane radius is given by 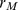, the helical polymer radius is given by 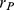, and 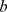 is the radial size of the enclosed polymer filament bundle.

In [11], a helical ansatz was employed for the conformation of the polymer bundle. However, for analytical calculational purposes this was assumed to reside inside an enclosing membrane tube *that remained perfectly cylindrical*, despite simulation snapshot evidence to the contrary [11]. Energetically stable ground state configurations were calculated for filopodia within the range of physiologically relevant parameters. However, due to the presence of very soft modes [14] for membrane tube deformations, it is unrealistic to analytically assume that the enclosing membrane tube will remain perfectly cylindrical. It would cost the membrane tube very little energy to deform in order to accommodate the enclosed helical fiber bundle (see Fig. 1). In order to calculate analytically the ground state configurations of realistic filopodia, and their corresponding energetic stability, we find that it is necessary to explicitly compute the conformation of the enclosing membrane tube. This is achieved by minimising a rigorously derived energy functional (defined below) that includes the elastic response of both the membrane and the fiber bundle while respecting the constraint that the helical polymer bundle must remain enclosed by the membrane tube.

## Results

Typical experimental parameter values for biological membranes range from [2] [15] 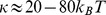, 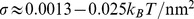. In order to compare the results of this work with that of [11], we take 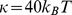 and 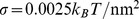 throughout in what follows. These values gives rise to a typical membrane tube radius of 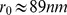. In order to estimate the radial size 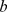 of a filament bundle, we consider a cross-section of 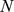 fibers each with a typical size 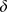, which we assume forms a hexagonally close packed structure [9]. A suitable continuum approximation for the bundle radius 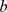 as a function of the number of fibers 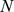 is then : 
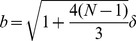
, which approximates to 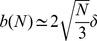, for 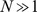. If we take 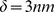 (for actin filaments), and 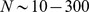, then typical biological fiber bundles possess radii 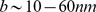. Thus we can see that the finite radius filament bundle considerations contained in this work become important for physiological filopodia.

The ground-state configuration of a filopodium is determined by finding the minimum of the total energy per unit length 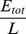 of Eq. (12) as given below. The relevant two parameters we need to minimise over are the 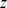 extension factor 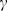, and the helical radius of the enclosed fiber bundle 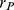, while keeping the number of fibers 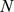 fixed.

**Figure 2:**
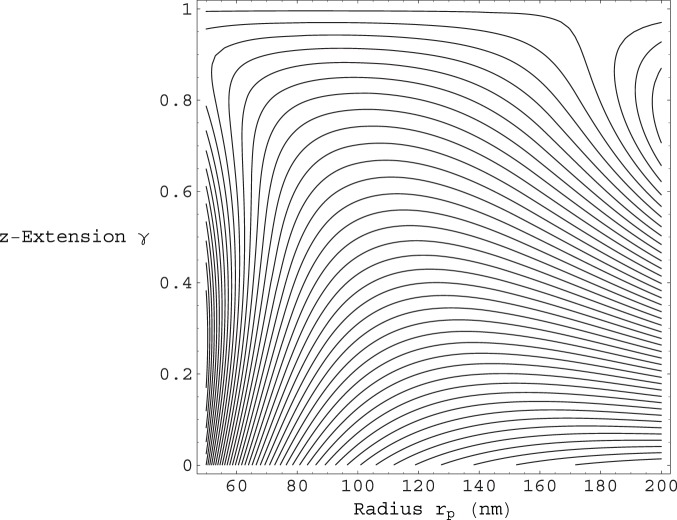
Contour plot of the total energy per unit contour length 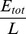 from Eq.(12). The energy is plotted as a function of the enclosed filament helical radius 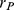, and the 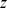 extension factor 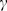. The membrane bending modulus is 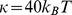 and the surface tension is 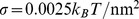. The same values of 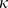 and 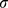 are used in all subsequent figures. The number of filaments in this case is given by 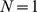. These parameters do not give rise to a local energy minimum. The contours near the top of the plot have values around 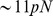, those contours near the middle 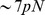, and the nearest to bottom contours on the plot 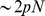, (at room temperature).

**Figure 3:**
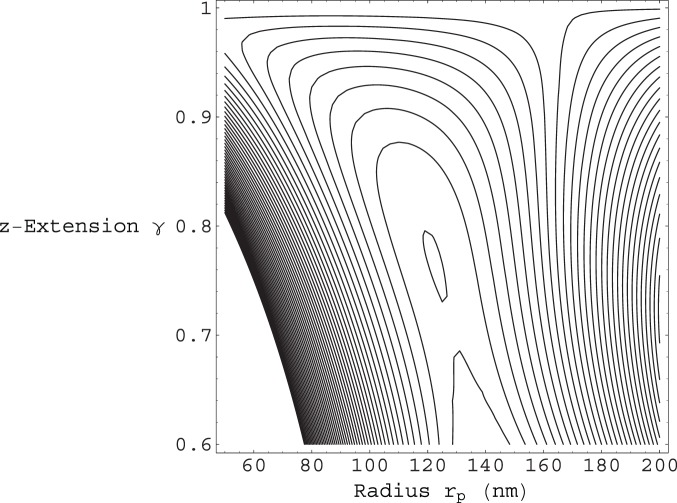
Contour plot of the total energy per unit contour length 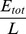 from Eq.(12). The energy is plotted as a function of the enclosed filament helical radius 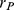, and the 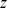 extension factor 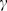. The number of filaments in this case is given by 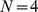. These parameters give rise to a local energy minimum at: 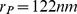 and 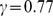, corresponding to one helical winding per 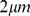 of contour length. Both the contours near the top and bottom of the plot have values around 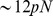, while the closed contour near the middle has a value of 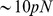.

**Figure 4:**
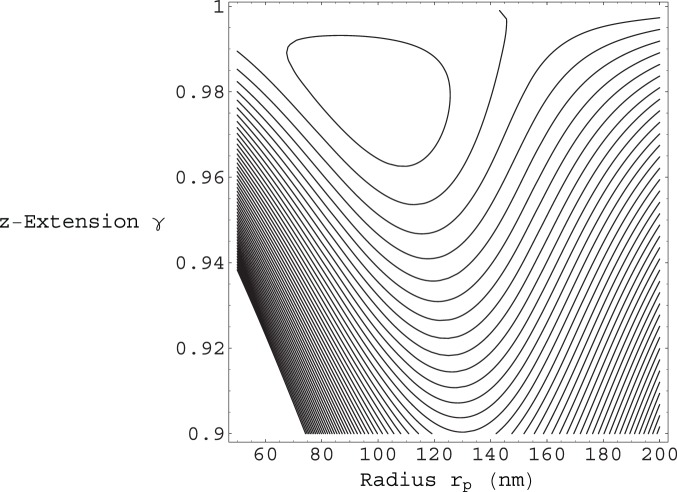
Contour plot of the total energy per unit contour length 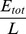 from Eq.(12). The energy is plotted as a function of the enclosed filament helical radius 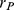, and the 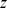 extension factor 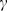. The number of filaments in this case is given by 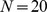. These parameters give rise to a local energy minimum at: 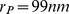 and 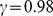, corresponding to one helical winding per 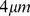 of contour length. The closed contour near the top of the plot has a value of 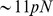, while the contours close to the bottom of the plot have values 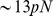.

**Figure 5:**
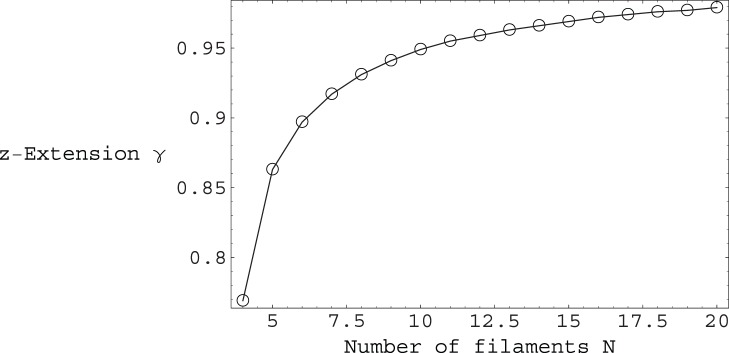
Plot of the extension factor 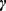 along the 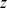 axis versus the number of filaments 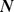. The 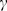 values plotted correspond to the energetic minima of the total energy per unit contour length 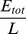 from Eq.(12), for a given number of filaments 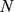.

**Figure 6:**
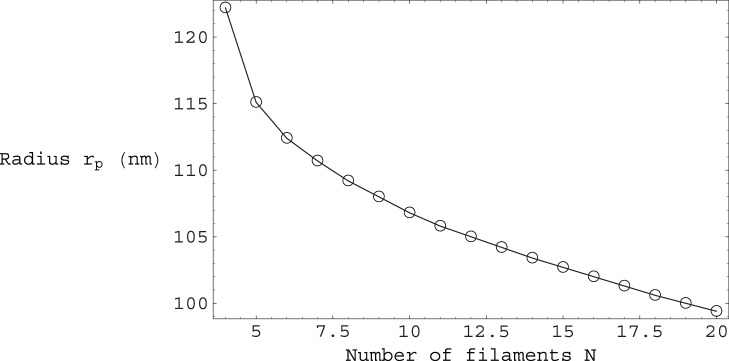
Plot of the polymer helical radius 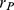 versus the number of filaments 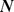. The 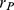 values plotted correspond to the energetic minima of the total energy per unit contour length 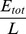 from Eq.(12), for a given number of filaments 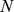. For comparison, note that 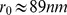.

**Figure 7:**
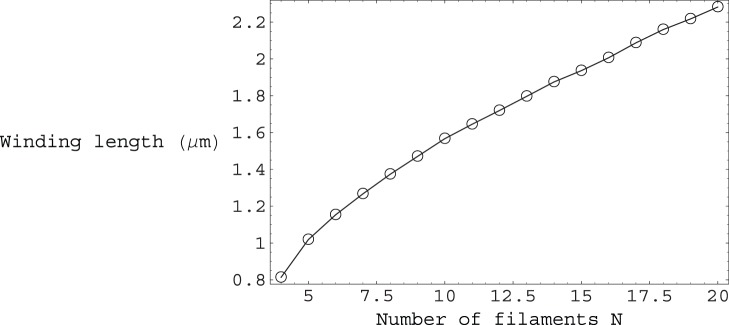
Plot of the polymer helical winding length versus the number of filaments 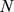. The winding length values plotted correspond to the polymer contour length required for one complete helical turn in order to maintain stability of the filopodium.

Shown in Fig. 2 is the contour plot of the total energy per unit contour length 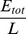 from Eq.(12). for a single fiber 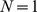. The energy is plotted as a function of the enclosed filament helical radius 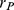, and the 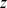 extension factor 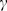. It can be seen from Fig. 2 that, for the 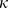 and 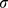 values used, a single fiber does not give rise to a local energy minimum, and is therefore unstable. We find that the minimum number of fibers required for filopodium stability is given by 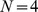, as shown in Fig. 3, which gives rise to a local energy minimum at: 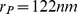 and 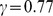, corresponding to one helical winding per 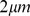 of contour length. From Fig. 4 we can see that for 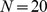 we have a local energy minimum at: 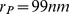 and 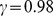, corresponding to one helical winding per 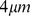 of contour length. Moreover, form Fig. 5 we can see that as the number of fibers 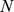 in a bundle increases, our filopodium remains stable, with the 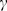 extension factor rapidly approaching the maximum allowed value of 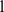. Furthermore, as 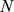 increases, we can see from Fig. 6 that 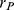 decreases, tending towards the limiting value of 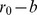, as the number of fibers becomes large. Additionally, we can see from Fig. 7 that the amount of helical winding required for stability reduces concomitantly also, as the number of fibers in a bundle 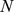 increases.

## Discussion

We have calculated theoretically the ground state configurations of filopodia, and found ‘islands of stability’ for typical filopodia within physiologically relevant parameters. Our calculation elucidates how experimentally observed filopodia can obviate the classical Euler buckling condition and remain stable up to several tens of 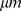 [1] [8] [13]. We find, as in [11] that the enclosing membrane tube tends to stabilise filopodia, rather than de-stabilise as a naive Euler buckling estimate might suggest.

The work presented here differs from that presented in [11] in the following, important ways. Firstly, we correctly incorporate the effects of a finite fiber bundle radial size 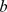, in this work, which was absent in [11]. Secondly, the total energy 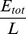 given by Eq.(12) of this work is calculated rigorously and analytically, taking into proper account the steric constraint of membrane tube enclosure of our semiflexible fiber bundle. The presence of soft modes for membrane tube deformations, implies that the membrane tube typically deforms in order to accommodate the enclosed helical fiber bundle, and does not remain perfectly straight, as analytically assumed in [11]. Thirdly, as we have found, there exists a delicate interplay between a fluid membrane tube and an enclosed semiflexible fiber bundle in filopodia. It is therefore imperative that the most accurate and correct total energy function for filopodia be calculated, as achieved in this work. Only then does it become possible to realistically describe the rather subtle issue of whether a given filopodium exists in a stable or a collapsed state. For example, we find in this work that the minimum number of fibers required for stability is given by 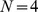, whereas in [11] all fibers with 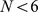 are deemed unstable.

Experimental observation of the results obtained in this work for the helical-like deformations of enclosing membrane tubes in filopodia would presumably be difficult. However, such helical membrane conformations are qualitatively supported by the snapshot pictures of simulation work carried out in [11], and could possibly be observed in the acrosomal reactions of the sea cucumber *Thyone* [8], and the horseshoe crab *Limulus* [16].

We adopt a ground state approximation in which thermal fluctuations are assumed to be small. Since the amplitude of these fluctuations is small at the high tensions of interest to us here, perhaps a few nm or less [17], this is a reasonable approximation.

Analogous steric constraints to those considered here are likely to be of relevance in other similar and important biological contexts, such as the packaging of semiflexible DNA in viral capsids, for example [18] [19] [20]. The interesting issue of mechanical stability in biological cellular tubes *without* an enclosed stiff polymer has also recently been considered in [21].

## Models

### Polymer Energy

In order to describe the filament bundle, inside filopodia, we study the semi-flexible polymer Hamiltonian 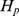 (where we chose energy units such that 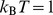 throughout):
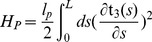
(1)with 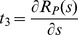. 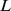 is the contour length of the fiber bundle, and we take the persistence length for un-crosslinked bundles of 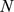 fibers to be 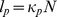 [12], where 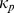 is the bending modulus of a single fiber (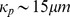 for actin [2]).

Any realistic deformation of the polymer must be able to pack a given contour length 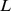 within a given radius and extension along the 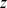 axis, as prescribed by the enclosing membrane tube. We therefore assume the most plausible conformation for the polymer as being that of a helix, as also outlined in [11].
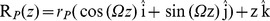
(2)


We have chosen to parameterise the polymer in terms of the 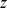 coordinate, as opposed to the arc-length 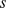, in order to simplify consideration of the required steric constraint between the polymer and the membrane as outlined below. Inextensibility for the polymer is maintained by requiring that:
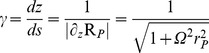
(3)


In this way we can easily translate between the arc-length 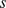, and 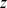 extension representations, by defining: 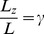 and 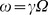, such that: 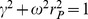.

The polymer part 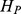 is thus straightforwardly calculated to be:
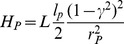
(4)


### Membrane Energy

In order to describe deformations of our membrane tube, we use:
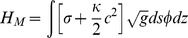
(5)where 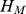 is the usual Hamiltonian for membrane elasticity [22] [23], containing both surface tension (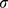) and rigidity (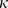) controlled terms.

We parameterise our membrane given by 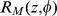 in the usual way as:
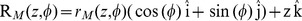
(6)


The membrane contribution 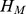 is calculated as follows. We proceed by writing perturbatively: 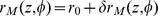, which involves the radial length scale 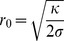. In this way we obtain:
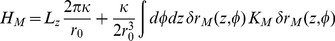
(7)where the kernel 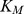 is given by: 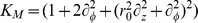.

### Steric Constraint

By inspection of Eqs. (2) and (6), we can see that the steric condition we need to apply to the membrane in order to guarantee polymer enclosure is given by:
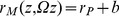
(8)where 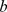 is the radial size of the polymer filament bundle. By writing perturbatively: 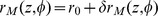, the steric constraint of Eq. (8) now implies: 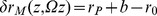. We enforce this steric constraint by introducing the following Hamiltonian 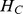:
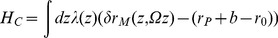
(9)which includes a Lagrange multiplier 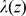 that ensures membrane tube enclosure of the confined polymer helix. While the steric relationship is strictly an inequality, on physical grounds the ground state polymer configuration always tends to contact the membrane because the longer the polymer the smaller the compressive load it can support before it buckles, becoming helical. Thus a long polymer will always tend to adopt a helical configuration, stabilised by the inward-pointing membrane force, at the maximum radius allowed by the steric constraint.

### Total Energy

In order to find the ground-state configuration of our filopodium, we need to find the conformation which minimises the total energy 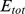 given by: 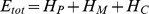. By varying 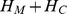 w.r.t. 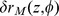 and 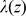, and by using the relevant Green functions, we obtain:
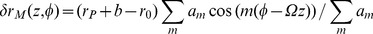
(10)along with 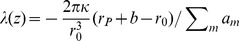, and where the Fourier coefficients 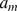 are given by: 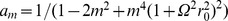. Note that an ansatz loosely similar to Eq. (10) was also used in [24] to minimise the energy for a stack of 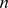 cylindrical membranes, in order to describe the helical coiling behaviour of myelin tubes. Indeed, the filopodia described in this work, consisting of a fiber bundle of radius 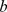 enclosed by a membrane tube, can analogously be thought of as an ‘
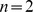
’ cylindrical membrane stack. In terms of the Fourier coefficients 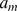, the membrane radius solution of Eq. (10) can additionally be seen to automatically satisfy the steric constraint: 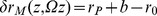.

Putting the result of Eq. (10) into 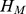 we get (valid to quadratic order in 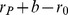):
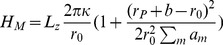
(11)


By inspection of the Fourier coefficients 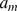, it can be seen that for small 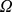 winding the leading order contribution to 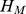 comes from the 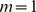 mode, and is proportional to 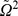. This leads to a relatively weak strength for the quadratic potential in 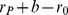, and is due to the fact that the 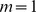, 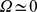 mode is an extremely soft mode for membrane tubes as shown in [14]. Indeed, the 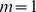, 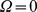 mode corresponds precisely to a rigid translation of the entire tube, and cannot therefore make any contribution to the membrane energy 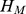. It can also be shown that the modes that contribute to 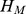 to next to leading order are the 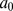 mode corresponding to a uniform dilation of the membrane tube, and the 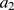 mode, which corresponds to a small deformation of the cross-section of our tube from a circular shape to that of an ellipse.

Utilising the inextensibilty conditions outlined above, we can easily re-write 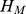 in terms of the contour length 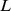, and the 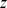 extension factor 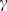. In particular we find for the winding rate 
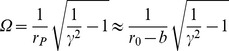
, to leading order. We thus arrive at our final expression for the total energy per unit length of a filopodium as (valid to quadratic order in 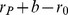):
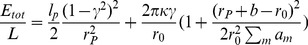
(12)where the Fourier coefficients 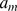 are now functions of 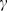. The ground-state configuration of our filopodium can now be determined by minimising the total energy per unit length 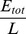 of Eq. (12), with respect to the two parameters 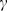 (the 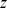 extension factor) and 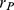 (the helical radius of the enclosed fiber bundle).
